# Epidemiological Studies on Porcine Trichinellosis in Five States of North East India

**Published:** 2019

**Authors:** Gohain Barua ACHEENTA, Chutia Pawan JYOTI, Raj HIMANGSHU, Sonowal DHARITREE, Rajkhowa UTTAM, Goswami CHANDRANI

**Affiliations:** 1. Department of Veterinary Public Health, College of Veterinary Science, Assam Agricultural University, Guwahati- 781022, Assam, India; 2. Department of Veterinary Biotechnology, College of Veterinary Science, Assam Agricultural University, Guwahati- 781022, Assam, India; 3. Department of Biosciences and Bioengineering, Indian Institute of Technology Guwahati, Assam, India

**Keywords:** Trichinellosis, Porcine, Sero-prevalence, India

## Abstract

**Background:**

This study was carried out to evaluate the epidemiological studies of trichinellosis in five states of North East India from Apr 2016 to Dec 2017.

**Methods:**

Overall, 865 different meat samples for detection of *Trichinella* larvae and 1580 sera samples for detection of anti-*Trichinella* antibody were collected. Intensity of infection with *Trichinella* larvae in meat was determined by HCL: Pepsin digestion procedure and anti-Trichinella IgG in serum were detected using excretory/secretory antigens, according to validated ELISA.

**Results:**

No *Trichinella* larva was detected by HCL: Pepsin digestion method. However, four (0.25%) samples were seropositive for *Trichinella* IgG and four inconclusive results as per cut off value. The highest seroprevalence was observed in Meghalaya (0.41%) followed by Assam (0.27%) whereas no seropositive cases were recorded in Arunachal Pradesh, Mizoram and Tripura.

**Conclusion:**

Trichinellosis is common in North East, India. However, it is suspected in communities where more than 75% of the population relish pork. Finally, there is a need for more research to establish the facts of trichinellosis in this region. Thus, public awareness, food hygiene, monitoring, and surveillance programme are suggested to implement for prevention of trichinellosis in this region.

## Introduction

*T*richinellosis is a zoonotic disease caused by nematode worms of the genus *Trichinella* (1) and occurs worldwide and infects all vertebrates including humans (2). Although infection with *Trichinella* is globally distributed (3), it has been documented rarely in India (4).

To date, nine species and three genotypes have been recognized within the *Trichinella* genus (5). The parasite has a direct life cycle with wide host range, which includes humans, pigs, wild boar (6), rats, horses, bear, walruses, some birds and reptiles (1). Several factors like socioeconomic background, cultural habits, political factors, geographic location, migration of humans and animals have a serious bearing on the epidemiology of trichinellosis in humans and animals (1,7, 8). Infection in pigs is perpetuated by swill feeding, eating infected rodent carcasses, tail-biting, infestation by faces from freshly infected animals or feeding on nonsterilized human food residuals (9). Farm management practices play an important role in the outbreak of trichinellosis in pig farms (10, 11).

The consumption of *Trichinella* spp. infected pork and wild boar meat are primarily responsible for human infections (12). To a lesser extent, human trichinellosis could result from the consumption of other domestic animals (e.g. horses, dogs, and sheep) and wild animals (e.g. walrus, bear, deer, etc.) (2). Synanthropic animals such as rats are regarded as an important reservoir of infection to the scavenging pigs.

Ingestion of the infected meat is known to be the sole route of transmission between infected and susceptible animals (13). Therefore, meat of domestic and wild animals must always be considered as a potential source of *Trichinella* infection to humans (14).

In India, human trichinellosis remained a neglected zoonosis (15) with sporadic cases until 2012 (16, 17). However, the presence of *Trichinella* has been conclusively shown by the different works of another investigator, who isolated *Trichinella* from cats, rodents and domestic pigs, respectively (18, 19).

However, there is a dearth of report regarding the epidemiological studies of porcine trichinellosis in northeast India. Hence, the present study was carried out to investigate the epidemiological status of trichinellosis in pigs in Assam, Meghalaya, Arunachal Pradesh, Mizoram and Tripura, five northeastern states of India.

## Materials and Methods

### Study design

This study was carried out on the pig population in five states of North East India from Apr 2016 to Dec 2017, which comprised of eight states. This region covers an area of 38076 km^2^ with the pig population of nearly 30 lakhs as per 19^th^ livestock census-2012 all India report (20).

### Sample collection

**Fig. 1:**
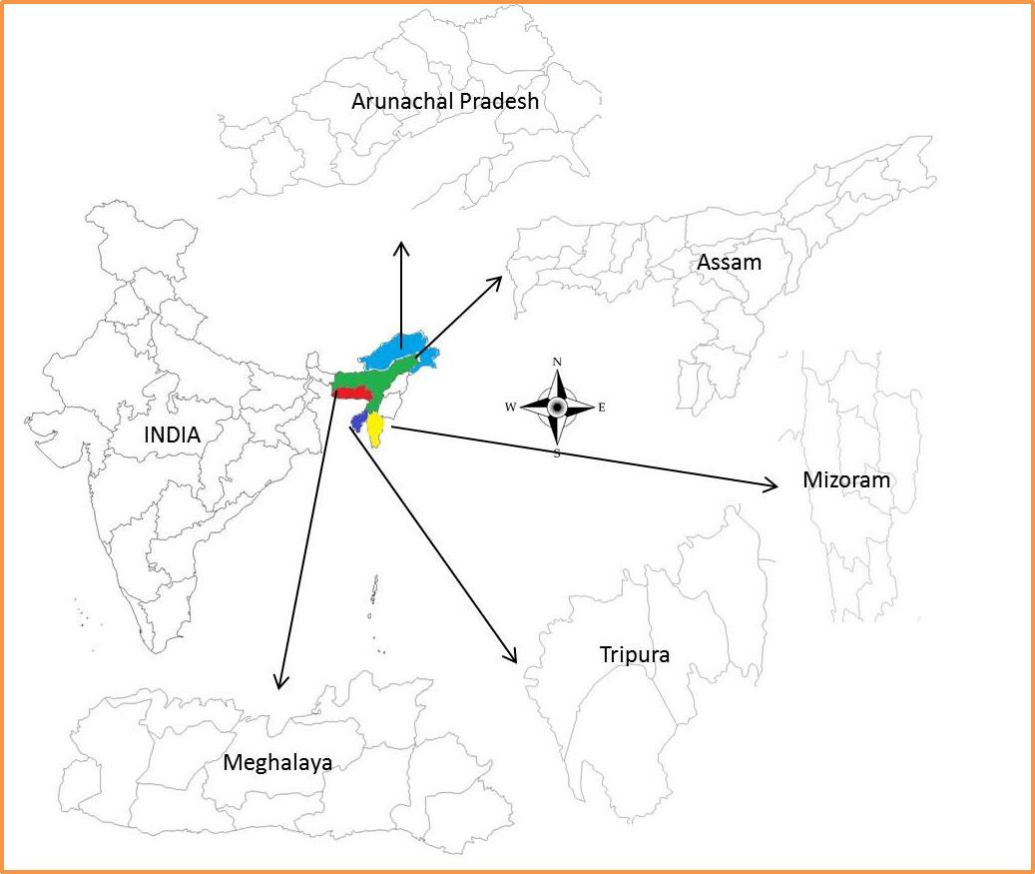
Map showing Assam, Meghalaya, Arunachal Pradesh, Mizoram and Tripura in North East India (Map not to scale)

Overall, 865 samples of lower jaw (masseter muscles), diaphragm (lumbar and coastal parts), tongue and filet (musculus longissimus dorsi) for detection of *Trichinella* larvae in meat and 1580 blood samples for detection of anti-*Trichinella* antibody were collected aseptically from Assam, Arunachal Pradesh, Meghalaya, Mizoram and Tripura (Fig. 1). The blood samples were collected in an evacuated tube, allowed to clot at room temperature and centrifuged at 1200 rpm for 10–15 min at room temperature. The serum was then transferred to 1.5 ml microcentrifuge tubes and stored at −20 °C until analysis.

### Detection of Trichinella larvae in meat

The intensity of infection with *Trichinella* spp. larvae in meat were determined by pepsin digestion procedure as per OIE recommendation (21). In brief, 100 g minced muscle sample (20 g per pig) was digested using an artificial digestive fluid consisting of 1% pepsin–HCl solution. If the larvae were detected, then individual pig tissue samples were to be separately digested. The larvae were further separated using the double separatory funnel method. Overall, 10 ml of sediment fluid was transferred into a gridded petri dish and examined under a stereomicroscope.

### Detection of anti-Trichinella antibodies in porcine sera

Porcine serum samples were tested to detect anti-*Trichinella* IgG using excretory/secretory antigens, according to validated in-house ELISA (22). The lyophilized *T. spiralis* excretory/secretory antigen gifted from International *Trichinella* Reference Centre, Rome, Italy was used as positive and negative controls.

## Results

### Trichinella larvae in different meat samples

**Fig. 2:**
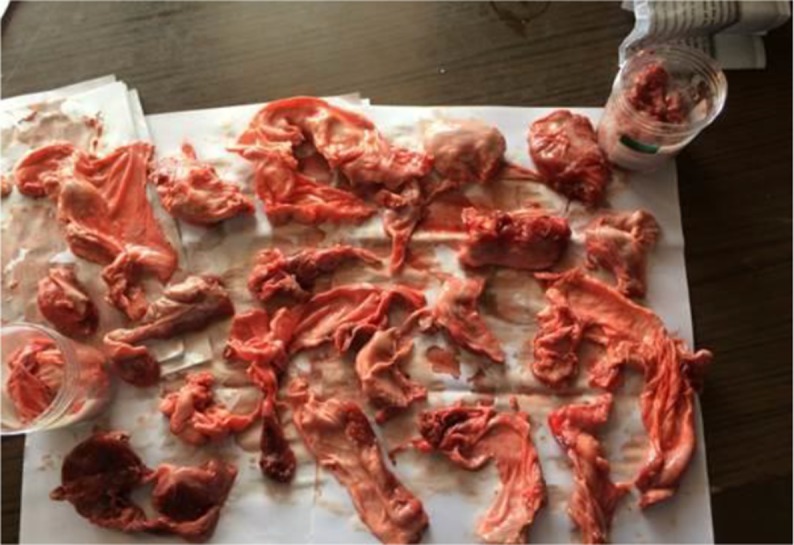
Tongue and diaphragm of pig collected from different meat markets and slaughterhouses

Different meat market and slaughterhouse survey were carried out for the collection of samples (tongue and diaphragm) (Fig. 2). No *Trichinella* larva was detected by HCL: Pepsin digestion method. For confirmation samples were again revalidated in Bombay Veterinary College, Mumbai, Maharastra and result were found similar.

### Seroprevalence of anti-Trichinella antibodies in porcine sera

Totally, 1580 pig serum samples were analyzed by ELISA for *Trichinella*. Overall seroprevalence was 0.25% and four inconclusive results as per cut off value (Table 1). The highest prevalence was observed in Meghalaya (0.41%) followed by Assam (0.27%). In Arunachal Pradesh, Mizoram and Tripura no seropositive cases were recorded. These samples were again revalidated in Bombay Veterinary College, Mumbai, Maharastra, and result were found similar.

**Table 1:** Sero-Prevalence rate of Trichinellosis in pigs of different stated/districts of North East India

***State***	***District***	***no. of pig serum***	***Sero Positive (%)***
	Kamrup	203	0 (0.00)
	Baska	77	1 (1.30)
	Nagaon	96	0 (0.00)
	Morigaon	78	0 (0.00)
Assam	Jorhat	88	0 (0.00)
	Sivsagar	53	0 (0.00)
	Dibrugarh	166	1 (0.60)
	Tinsukia	172	0 (0.00)
	North Lakhimpur	81	0 (0.00)
	Dhemaji	112	1 (0.89)
	Total	1126	3 (0.27)
	Jayantia Hills Division	124	1 (0.81)
Meghalaya	Khasi Hills Division (Ribhoi, East & West KH)	118	0 (0.00)
	Total	242	1 (0.41)
	Subansiri	61	0 (0.00)
Arunachal Pradesh	Papum pare	54	0 (0.00)
	Total	115	0 (0.00)
	Aizawl	30	0 (0.00)
Mizoram	Kolasib	25	0 (0.00)
	Total	55	0 (0.00)
	West Tripura	30	0 (0.00)
Tripura	Khowai	12	0 (0.00)
	Total	42	0 (0.00)
Total		1580	4 (0.25)

## Discussion

The seroprevalence of trichinellosis in North East States of India was observed as 0.25%, whereas *Trichinella* larvae were not detected using pepsin–HCl digestion method. This might be due to fact that specificity of excretory-secretory (ES) antigen-based indirect ELISA has been reported to vary from 90.6% to 99.6% (23) or it may be due to low level of infection in seropositive pigs.

Moreover, the highest prevalence was observed in Meghalaya (0.41%) followed by Assam (0.27%). Findings of this study were different than that of the previous study where 38 pig serum samples were collected from Assam but none of the samples were found positive for *Trichinella* (4).

Many authors have used indirect ELISA for estimating sero-prevalence of trichinellosis (24, 25). The previous study reported a prevalence of 0.47% with western blot (26) and 1% with antibody ELISA on pig’s sera in Kathmandu, Nepal (27). Both studies suggest serological evidence of trichinellosis.

Previous studies indicate a low prevalence of swine trichinellosis in India. Studies conducted on slaughtered pig carcasses to isolate *Trichinella* spp. have revealed prevalence rate ranging from 0.4%–0.6% (18, 19). There are no reports of Trichinellosis from states like Bombay and Madras associated with swine carcasses (28, 29). In contrast, examination of pig diaphragm samples from Deonar abattoir, Mumbai by PCR assay revealed the prevalence of trichinellosis as 0.69% (30). Similarly, in another study, the prevalence of Trichinellosis using acid-pepsin digestion, PCR and ELISA were recorded to be 0.27%, 0.27% and 2.69% respectively (31).

The parasite is still a health and food safety problem for countries worldwide (32). As per Chapter 8.16 of the OIE Terrestrial Animal Health Code for the importation of meat or meat products of domestic pigs, pork must be tested negative for Trichinella larvae or has to be processed to ensure the inactivation of Trichinella larvae in accordance with the recommendations of the Codex Alimentarius.

Human trichinellosis outbreaks following consumption of raw or undercooked wild boar meat were reported from the Uttarakhand state of India, where 11 deaths occurred from 70-suspected cases (33). Epidemiological studies in pigs in north India are required and the infection is likely to be under-diagnosed, and it is necessary to explore the existence of the parasite among domestic and wildlife reservoirs.

## Conclusion

This study will be useful to cover the missing epidemiologic gaps related to porcine trichinellosis in North East India where more than 75% population relish pork as a protein supplement in cheaper rate. Trichinellosis remains a rare zoonosis in North East India. However, we recommend strict monitoring and surveillance programme to generate scientific baseline data to support pig industry of this region. Furthermore, this research will also help in capacity building workshop for detection of trichinellosis in large pig consumer. Widespread studies must be carried out in wild animals like walrus, bear, deer, wild hog, etc. to recognize the reservoir hosts in this part of the country.
